# Supplementary material for: Control of endothelial cell polarity and sprouting angiogenesis by non-centrosomal microtubules
Source: eLife. 2018 Mar 16;7:e33864. doi: 10.7554/eLife.33864 (PMC5898915; doi:10.7554/eLife.33864)
Supplement: Transparent reporting form [file elife-33864-transrepform.docx]

***eLife’s* transparent reporting form**

We encourage authors to provide detailed information *within their submission* to facilitate the interpretation and replication of experiments. Authors can upload supporting documentation to indicate the use of appropriate reporting guidelines for health-related research (see [EQUATOR Network](http://www.equator-network.org/%20)), life science research (see the [BioSharing Information Resource](https://biosharing.org/)), or the [ARRIVE guidelines](http://www.plosbiology.org/article/info:doi/10.1371/journal.pbio.1000412) for reporting work involving animal research. Where applicable, authors should refer to any relevant reporting standards documents in this form.

If you have any questions, please consult our Journal Policies and/or contact us: [editorial@elifesciences.org](mailto:editorial@elifesciences.org).

**Sample-size estimation**

- You should state whether an appropriate sample size was computed when the study was being designed
- You should state the statistical method of sample size computation and any required assumptions
- If no explicit power analysis was used, you should describe how you decided what sample (replicate) size (number) to use

Please outline where this information can be found within the submission (e.g., sections or figure legends), or explain why this information doesn’t apply to your submission:

No explicit power analysis was used. The sample size was chosen based on common practice in the field for each specific experiment and based on the uniformity of the readout within and between experiments. This is indicated in Materials and Methods.

**Replicates**

- You should report how often each experiment was performed
- You should include a definition of biological versus technical replication
- The data obtained should be provided and sufficient information should be provided to indicate the number of independent biological and/or technical replicates
- If you encountered any outliers, you should describe how these were handled
- Criteria for exclusion/inclusion of data should be clearly stated
- High-throughput sequence data should be uploaded before submission, with a private link for reviewers provided (these are available from both GEO and ArrayExpress)

Please outline where this information can be found within the submission (e.g., sections or figure legends), or explain why this information doesn’t apply to your submission:

All the information about the number of experiments and the number of measurements can be found in the corresponding figure legends of all plots shown in the figures.

**Statistical reporting**

- Statistical analysis methods should be described and justified
- Raw data should be presented in figures whenever informative to do so (typically when N per group is less than 10)
- For each experiment, you should identify the statistical tests used, exact values of N, definitions of center, methods of multiple test correction, and dispersion and precision measures (e.g., mean, median, SD, SEM, confidence intervals; and, for the major substantive results, a measure of effect size (e.g., Pearson's r, Cohen's d)
- Report exact p-values wherever possible alongside the summary statistics and 95% confidence intervals. These should be reported for all key questions and not only when the p-value is less than 0.05.

Please outline where this information can be found within the submission (e.g., sections or figure legends), or explain why this information doesn’t apply to your submission:

All data are shown using box plots where rectangles represent the second and third quartiles, contain a line corresponding to the median value and are extended with whiskers showing the minimum and maximum, except in Figure 6 – Figure Supplement 1B and Figure 9D, which depict mean ± SEM. In Figure 1 – Figure Supplement 1B and Figure 4 – Figure Supplement 1B, the mean value ± SEM is indicated within the pictures. This is clearly indicated in the figure legends as well as in Materials and Methods. In addition, the statistical tests used, the number of measurements and of independent experiments are indicated in the figure legends. For all statistical tests we reported the range in which p values felt using Prism5 conventions (based on a number of “*”, indicated in the figures) in all figure legends.

(For large datasets, or papers with a very large number of statistical tests, you may upload a single table file with tests, Ns, etc., with reference to sections in the manuscript.)

**Group allocation**

- Indicate how samples were allocated into experimental groups (in the case of clinical studies, please specify allocation to treatment method); if randomization was used, please also state if restricted randomization was applied
- Indicate if masking was used during group allocation, data collection and/or data analysis

Please outline where this information can be found within the submission (e.g., sections or figure legends), or explain why this information doesn’t apply to your submission:

No explicit randomization method was used as no clinical experiments were done but the different experimental groups were treated with comparable conditions (endothelial cells from the same batch and zebrafish embryos from the same line, same culture and raising condition and analyzed at the same time)

Masking was not used during analysis. This is indicated in Materials and Methods.

**Additional data files (“source data”)**

- We encourage you to upload relevant additional data files, such as numerical data that are represented as a graph in a figure, or as a summary table
- Where provided, these should be in the most useful format, and they can be uploaded as “Source data” files linked to a main figure or table
- Include model definition files including the full list of parameters used
- Include code used for data analysis (e.g., R, MatLab)
- Avoid stating that data files are “available upon request”

Please indicate the figures or tables for which source data files have been provided:

An Excel sheet with the numerical data represented in each graph is provided for each figure and figure supplement as source data.
